# Tumor cell-free DNA detection in CSF for primary CNS lymphoma diagnosis

**DOI:** 10.1186/s40478-019-0692-8

**Published:** 2019-03-18

**Authors:** V. Rimelen, G. Ahle, E. Pencreach, N. Zinniger, A. Debliquis, L. Zalmaï, I. Harzallah, R. Hurstel, I. Alamome, F. Lamy, J. Voirin, B. Drénou

**Affiliations:** 1 Laboratoire d’Hématologie- Groupe Hospitalier de la Région de Mulhouse Sud-Alsace, Hôpital E. Muller, 20 avenue de Dr Laennec, 68100 Mulhouse, France; 2 Service de Neurologie, Hôpitaux Civils de Colmar, 39, Avenue de la Liberté, 68000 Colmar, France; 3 Laboratoire d’Oncobiologie, Centre Hospitalier Universitaire de Strasbourg, Hôpital de Hautepierre, 1 avenue Molière, 67000 Strasbourg, France; 4 Laboratoire d’Hématologie et d’Hémostase, Hôpitaux civils de Colmar, 39, Avenue de la Liberté -, 68000 Colmar, France

To the editor,

Primary central nervous system lymphoma (PCNSL) is a rare disease accounting for around 3% of primary CNS tumors. Its diagnosis is usually based on cranial MRI and brain biopsy (including immunophenotyping for faster diagnostic confirmation [4]). The identification of lymphoma cells in the cerebrospinal fluid (CSF) or vitreous fluid by cytology (generally associated with flow cytometry) in association with typical neuroimaging allow faster and less invasive diagnosis. PCNSL characterization, frequently leads to diagnosis of diffuse large B-cell lymphoma (DLBCL), belonging to the ABC subgroup [3]. Moreover, MYD88 mutations are detectable in 58 to 76% of PCNSL cases, in about 30% of ABC DLBCL patients, and in the majority of lymphoplasmacytic lymphoma cases [6, 7, 11, 13]. Since this mutation is not described in glioblastoma or in other solid metastatic tumors, its detection in the cerebrospinal fluid (CSF) [10] could be helpful for PCNSL diagnosis without invasive surgical biopsies, such as IL10 concentration [12] and microRNA profiling [1]. The MYD88 L265P mutation detection in cell DNA from vitreous aspirates [2] and CSF [10] was reported to improve the PCNSL diagnosis. The aim of our study was to evaluate the contribution of cell-free (cf) DNA from the CSF with a valuable molecular tool detecting the tumor-specific mutation MYD88^L265P^, using ddPCR in known MYD88^L265P^ PCNSL.

**Fig. 1:**
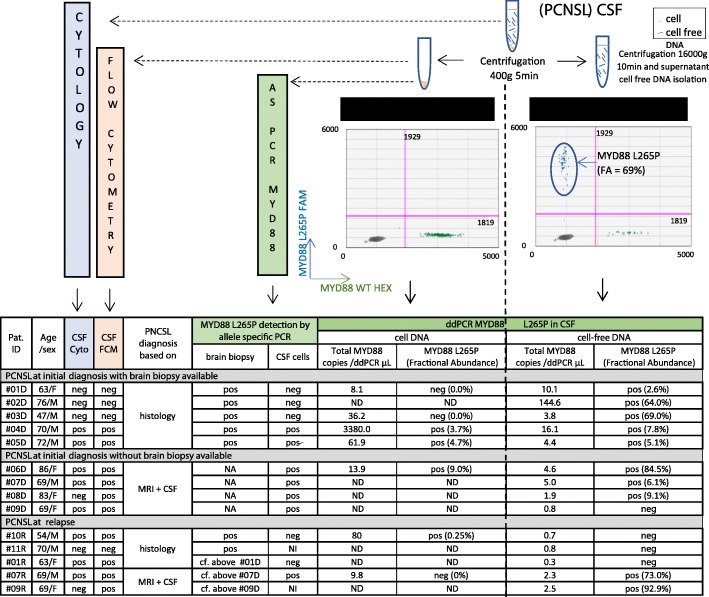
MYD88 L265P quantification by ddPCR. Technical workflow for the CSF analysis and 2D ddPCR diagram of the fluorescence amplitude. Lower left quadrant contains the droplets with no MYD88 alleles; upper left contains droplets with MYD88^L265P^ cfDNA; upper right contains droplets with both wild-type and mutant alleles; lower right contains droplets with MYD88 wild-type DNA. ddPCR results table for cell and cell-free CSF and brain biopsy, comparison with cytology and FCM. NA, not available; ND, not determined; NI, not interpretable

This retrospective study was conducted between August 2016 and June 2018 on a series of 11 MYD88^L265P^ PCNSL patients without ocular infiltration. The MYD88 mutation status was established either on brain biopsy (*n* = 7) or in cell DNA from CSF (*n* = 4) with an allele specific (AS) PCR technique. CSF samples at initial diagnosis (*n* = 9) or relapse (*n* = 5) were processed within 4 h after lumbar puncture. After CSF centrifugation (Fig. 1), the cell pellet and the previously discarded supernatant (1.5–5 mL) were collected for cfDNA isolation and ddPCR for the detection of the NM_002468:exon5:c.T778C(p.Pro265Leu) MYD88 variant. Sensitivity thresholds were established by a dilution study with the lower limit of quantification and detection found to be 0.9 copy/μL and 0.2 copy/μL, respectively. The specificity was evinced by the absence of L265P-positive droplets in 10 CSF samples from nonlymphomatous lesions.

The presence of cfDNA was detected in PCNSL CSF with a median value of 3.1 cfDNA copies/μL ddPCR mix (Fig. 1). Substantial variations of the amount of cfDNA were observed and four cases exhibited less than 1 copy/μL ddPCR mix, even though special care was given to the parameters affecting the quantity and quality of cfDNA, such as pretreatment delay, sufficient CSF volume, DNA isolation process and storage. The MYD88^L265P^ mutation was detectable in 10 out of 14 cell-free CSF samples, and not in the four cases with less than 1 copy wild-type MYD88/μL. In these samples, MYD88^L265P^ was detected in the CSF cell DNA using ddPCR only (#10R) or AS PCR (#09D). The MYD88^L265P^ detection rate in CSF combining both CSF fractions achieved 86% (12/14 cases). Two cases, at relapse, remained negative for MYD88^L265P^ detection in CSF, most probably due to a low cfDNA input or possible clonal evolution. The median fractional abundance (FA) was 7%, varying from 2.6 to 92.9%. FA was higher than in previous studies using plasma [5, 9], probably because CSF directly bathes the brain tumor, without background hematopoietic DNA retained by the blood–brain barrier. Furthermore, mutated cfDNA FA was higher than in the cell pellet DNA in six out of seven available samples. Moreover, in three cases (#01D; #03D; #07R), the L265P variant could only be detected in the cell-free fraction. Finally, cfMYD88^L265P^ was present in the absence of lymphoma cells using cytology and flow cytometry (FCM) in three cases at diagnosis (#01D; #02D; #03D), as it was described in recurrent/refractory CNS lymphoma [8]. Even if hot spot mutation is predominant in PCNSL, our cost-effective, highly sensitive ddPCR approach is limited to a restricted number of mutations and will miss PCNSL bearing other mutations.

This is the first report comparing cell and cell-free tumor load in CSF from PCNSL, showing the contribution of cell-free tumor detection in CSF for diagnosis. This study shows that detection of tumor cell and cell-free DNA is feasible using a workflow combining FCM and molecular biology. Moreover, ddPCR could be used for the tumor characterization of actionable mutations and longitudinal monitoring of the disease. We anticipate that this technique might also be applicable to other brain tumors with known hotspot mutations.
